# Normalcy, Race, and Biomedical Technology in Brazil: The Case of Low White Blood Cell Count

**DOI:** 10.3389/fsoc.2019.00075

**Published:** 2019-11-29

**Authors:** Elena Calvo-González

**Affiliations:** Postgraduate Programme in Social Sciences, Federal University of Bahia, Salvador, Brazil

**Keywords:** race, race relations, biomedical technology, Brazil, leukopenia

## Abstract

This article explores the intersection between low-complexity biomedical technologies and ideas about race in Brazil. Using ethnographic material collected in the northeastern city of Salvador on the clinical management of low white blood cell count (leukopenia), and debates involving doctors, biomedical scientists, and social movement activists on establishing racialized parameters in complete blood count tests, I explore how the notion of normalcy is connected to ideas about racial difference. Taken both at a population and individual bodily level, normalcy serves to contrast local history, portrayed as the result of widespread admixture between groups, with other national contexts, such as that of the United States. While a material body that cannot be classified as racially pure is seen as normal in the contemporary Brazilian context, nevertheless these pure racial types feature discursively as existing in a long lost past of Brazil's history. At the same time, normalcy can also be locally challenged by certain actors, such as social movement activists, who underline the specific experience of certain racialized bodies, questioning the overarching national narratives of admixture and arguing for the need to recognize these bodies as normal as well, particularly in the context of political struggle for the reduction of social inequalities. These two ways in which normalcy appears articulated with local meanings of race gives way to seemingly contradictory and confronting discourses. Thus, racial categories that are explicitly not identified with admixture are seen as a thing of the past (the history of the Nation) and a thing of the future (a more racially equal country). This can be better comprehended by the process through which different historical discourses on racial difference in Brazil appear in connection with, or as a proxy for, ideas such as nation, population or gender, originating from different places and moments in history.

## Introduction: Race, Normalcy, and the Measuring of Bodies

October 2006. I am at the regional hematological clinic in the northeastern Brazilian city of Salvador da Bahia, sitting in the corner of the blood sampling ward, where assistant nurses take blood samples from patients to be sent for analysis. A man in his late twenties/early thirties enters the room. He seems to be a regular patient, since he greets all the assistant nurses by first name. Before he sits down on the extraction chair, one of the nurses asks him if he's done his “round the block run” already. He nods, takes a seat and extends his left arm while holding his fist tight. She proceeds to take the blood sample while chatting vivaciously with him. After the blood sample had been collected, the patient leaves, while the assistant nurse puts the blood vial on a tray for collection. I ask her what she meant by the “round the block run,” wondering if it was part of the common banter of exchanging minor details about each other's lives that frequently characterizes everyday social interactions in Bahia. The nurse responded: “it's because he's Black, and we know that if he doesn't do that (run around the block) his lab results for his white blood cell count would come back outside the normal range.” She explained that some Black individuals naturally had a lower white blood cell count, but that the reference values that laboratories used meant that their blood tests always came back highlighted as abnormal, when in fact it was a normal condition of their bodies and posed no health threat. They asked these patients to take a brisk walk or run around the block because empirically they had realized that this raised their white blood cell levels enough so that the values fell within normal ranges^1^.

In spite of having been disavowed by mainstream genetics, race has proven to be an enduring category to characterize and classify bodily differences, both in everyday life as well as in diverse specialized fields, such as biomedicine. As anthropologists working with race and biomedicine, we should be aware of the powerful role that biomedicine has in re-defining “truths” about what is normal and what is pathological about bodily processes. This is particularly relevant in the context of the ever-growing popularization of low-cost biomedical technologies. These technologies, through objectively “reading” bodies and comparing them to a standard of normalcy, powerfully set new parameters and truths about bodily difference. At the same time, we should be attentive to how these truths often co-exist with other readings of bodily difference. These other ideas include ideas about race, often having multiple meanings and disguised in related ideas of bodily difference, be it in terms of individuals or populations (M'charek, 2013). This multiplicity of notions about difference often results in ambiguous and at times conflicting narratives regarding the link between race and disease, with other categories, such as nation or population, appearing intertwined. This very adaptability and capability to incorporate other ideas about difference has been pointed out as being central to understanding the enduring presence of race, both in biomedicine and in everyday life (Pollock, 2012).

In this article, I want to explore how the notion of normalcy in biomedicine, by facilitating the association of general ideas about difference and race, can contribute to the durability of race as a classificatory concept for human diversity. Using ethnographic material drawn from the northeastern Brazilian city of Salvador, I explore how ideas about normalcy are invoked in the local use of low-complexity diagnostic biomedical technology, using as a case study the measuring of white blood cell levels within a complete blood count. As several authors, notably Canguilhem (1989), have reminded us, normalcy, a statistical norm defining a pathology or a deviation for which a medical intervention should be applied, is necessarily embedded in its social context, from which it cannot be separated. Hence, for Canguilhem, health itself must be defined within the specific context of an individual and her relation with the environment in which she lives.

The different notions that the concept of normalcy encompasses, of which Canguilhem reminds us are those such as ideal, average, and common type, were also central in how ideas about race were crafted in 19th century scientific racism. Each racial group was seen as having a common type, with the features that defined each of them obtained through measuring characteristics such as skull size and shape, nose width and length, lip and teeth size, and other bodily features such as arm to body length ratio. The average measurements of these features for each group was established and then compared to the average for “whites,” within an *apriori* established racial hierarchy in which the white, heterosexual male^2^ was seen as the ideal for humanity, inherently superior to other groups.

Scientific racism was also already permeated by the idea that the deviation of other races from the white norm expressed itself and, in turn, could be gauged by the varying prevalence and expression of diseases, be they physical or mental (Stepan, 1991). Measuring and comparing bodies to established ideals of racial normalcy was incorporated into biomedicine's everyday practices, in processes that entangled notions of heredity, population, and nation^3^. Physical features such as lung capacity (Braun, 2014), pain threshold, blood conditions (Wailoo, 1999), or mental traits, amongst others, were measured from samples from within racial groups and then compared between “whites” and racialized “others.” While the similarities between groups were overlooked, the differences, when found, were identified as having a biological origin, helping to establish the idea that there were underlying natural differences between the races. This association would prove persistent, and even when the hierarchical aspect of race was eventually abandoned, its use as a legitimate concept to characterize and classify human biological diversity was maintained.

Most biomedical diagnostic technologies provide objective measurements of bodily parameters, that are then set within scales of normal and abnormal. Some of these parameters can be directly or indirectly racialized, both when establishing different reference values for certain groups and in biomedical discourses regarding morbidity and choice of treatment. In this process of racialization, normalcy appears not only related to an ideal about what a healthy body should be, but also to narratives about the nation and its population history, thus easily taking over or, at the very least, acting alongside other ideas about bodily difference. By analyzing ethnographically how complete blood count, a relatively simple, cheap, and widely used technology, mobilizes and (re)enacts notions of race, I aim to contribute to a wider field of anthropological studies about biomedicine and technology.

## Local Histories of Race in Brazil

Brazil's history of race relations has certain particularities of which the reader should be aware before I go further into the ethnographic details. With a much smaller European population when compared to other colonies, such as British enclaves, Portuguese settlers soon started to establish informal unions, first with indigenous women, and later on with the enslaved workforce brought to replace indigenous slavery. The result of this process was the widespread occurrence of mixed-unions, with their offspring often being incorporated into the slavemasters' houses, raised away from the slavequarters.

With the independence of the country and the need to establish a project for the nation in-the-making, which coincided with the formulation and spread of scientific racism in Europe and the United States, the present and future racial composition of the country became a heated topic of debate amongst the country's intellectuals and politicians. While the miscegenation of Brazil's population was taken as a fact, most of its intellectuals thought of Brazil as having a natural “tendency” of becoming a European-like country. International discourses of scientific racism propounding the superiority of the white race and the evils of miscegenation were interpreted and reshaped by Brazilian intellectuals to fit the local history of widespread mixed unions. Some authors, such as Nina Rodrigues, argued that Brazil would achieve a white future population by stopping the influx of enslaved Africans, seen as less eugenic than “whites,” substituting them with European immigrants. Other intellectuals, such as Sílvio Romero or João Batista de Lacerda, argued for the possibility of the progressive whitening of the population through intermarriage between the already existing population, seen as admixed, and incoming European immigrants. Trying to address these debates, the state drafted policies stimulating European immigration to substitute the enslaved workforce after abolition of slavery and progressively “whiten” the country's population, either through full replacement or progressive admixture between immigrants and the local population.

The first Brazilian population census, dating from 1872, reflected this process, registering *pardo* (which can be loosely translated as “brown”) and *caboclo* (mixed indigenous ancestry) as intermediate categories between *branco* (white) and *preto* (Black) (Piza and Rosemberg, 1999). The arrival of over five million European immigrants through State programmes or through personal networks was nevertheless not enough to achieve the projected “whitening” of the country. This realization, alongside the impact of cultural currents such as vanguard modernism, that celebrated local cultural specificities, facilitated a shift in intellectual discourses in the 1930s. This was particularly so with the publication of Gilberto Freyre's Masters and Slaves in 1933. Freyre considered miscegenation as the key defining feature of the country and its population. The historical experience of Portuguese settlers with mixture in their own country and other colonial enterprises meant, according to Freyre, that social relations between masters and slaves, although still conforming to the patriarchal system of domination imposed by the Portuguese colonists, also presented a degree of social proximity which facilitated close contact, including sexual, between these two groups. The vision that Brazil was a country with relatively congenial race relations^4^, and that it did not suffer from a “race problem” when compared to other countries, was highly influenced by this interpretation of its history, as well as by the benign terms in which Freyre described Portuguese slavery.

After the horrors of World War II, a UNESCO project was set up to explore whether Brazilian society could be taken as a model for harmonious coexistence between different groups. The studies from within this country-wide research project, led by anthropologists and sociologists, focused on topics such as the relative inter-class mobility of colored people, the absence of a clear distinction separating “whites” from “blacks” and the assimilation of the latter into mainstream society (Pierson, 1971), or the documentation of how the existence of several intermediate racial categories maximized ambiguity (Harris, 1956). So was the contrast between the US system of race relations, based on an evaluation of ancestry that places anyone with any degree of African ancestry in the Black group, with the Brazilian system, in which an individual's race is based on an assessment of the absence or presence of physical traits deemed to be of non-European origin, such as skin color, hair texture or facial features, alongside markers of social status such as educational level, demeanor, or clothing. In this manner, two people with a similar physical appearance could be classified into different racial categories, with the person holding higher status marks being classified in a lighter category, with social status offsetting, to a certain extent, the presence of physical features seen as marks of African ancestry (Nogueira, 1955).

Some of the research projects developed under the UNESCO initiative reached conclusions that directly contradicted the depiction of Brazil's race relations in idyllic terms, uncovering the existence of racism and racial prejudice, or the strong correlation between class position and race (Pinto, 1953). However, although there were already initiatives against racism and embryonic forms of organized black social movements throughout the early 20th century, these would increase in number and activity during the political “opening up” in the last years of the military dictatorship, and after the Promulgation of the 1988 post-dictatorship Constitution, in which racism was criminalized. The condemnation of persistent racial inequality and racism within Brazilian society would intensify in the 1980s, both within activist and academic circles. Extensive academic analyses of the statistical link between social indicators of well-being and wealth showed that figures for the intermediate census group of “browns” (*pardos)* were actually closer to those of “blacks” (*pretos)* (Hasenbalg, 1979) than to “whites.” This was interpreted as challenging Degler's (1971) “mulatto escape hatch” hypothesis of the privileged position granted to individuals placed in the intermediate racial category. Organized social movements, siding with Degler's arguments, also highlighted how the ideology of Brazil as a racially admixed and harmonious country meant the weakening of a stronger Black identity. Intermediate “racial” categories were, according to this line of thinking, central to the ideological valuing of whiteness, helping to defuse solidarity between darker and lighter non-whites. The reworking of a shared experience of discrimination, highlighted by statistical indicators, would imply the strengthening of a common identity as “blacks” (*negros)* that would encompass all those who self-identified as “non-whites” (Guimarães, 1999).

After the 2001, Durban conference and the acknowledgment by the Brazilian government of the existence of racism in the country, several tiers of government at the municipal, regional state, and federal levels designed and implemented affirmative action policies to reduce racial inequalities. While the need to reduce these inequalities was, at the time, accepted by most actors involved in public debates, the adoption of the amalgamated category *negro* in the implementation of these policies, for example to identify beneficiaries for university entry quotas, was rather controversial^5^. Besides some general criticism on the use of the category race by the State, in a country where there had been no racial segregation laws, other more specific issues were raised, such the hands-on use of such a category, examining its practical use in a country where there is no consensus on where the demarcation between “whites” and other racial categories stands^6^. In the case of affirmative action in health, the field of so-called Black Population Health, a related concern was that these policies would encourage the reification of race as a biological category. The explicit link made between certain conditions, whether genetic (such as sickle-cell disease) or multifactorial (such as hypertension), and “blackness” could contribute, according to some authors (Fry, 2005), to naturalize race as related to the realm of genetics and inherited biology.

## Race and Normalcy in Salvador, Bahia

Bahia is often seen as one of the “blackest” states in Brazil, due to the long history of slavery, the high influx of enslaved peoples, and the relatively limited presence of European immigrants (particularly when compared to Southern and Southeastern states). This is particularly clear in Salvador, where I did my fieldwork. With almost 80% of its population self-identifying as Black or “brown,” its African influenced local cuisine, a strong presence of Afro-Brazilian religious communities, and numerous other expressions of African influenced culture, such as Black cultural associations, and carnival ensembles, the capital of the state of Bahia, and nearby towns in the Recôncavo region, are often taken to be repositories of Blackness and Black culture within Brazil (Santos, 2005). This context certainly affects how race is done locally, for example in the higher number of people, particularly younger generations, who self-identify with the term *negro* (Sansone, 1996), or in the type of physical and status features that lead to an individual being recognized as white. Some individuals that are locally read as “whites” would be considered “brown” in other regions of the country, particularly in regions located to the south of Rio de Janeiro. The circulation of national and international black social movement activists, the influence of global youth culture and the effects of the tourism industry are all factors that have been analyzed as influencing how locals “do” and understand race (Sansone, 2004).

I started my fieldwork in Salvador in the early 2000s, to assess the impact that affirmative action policies in the field of health had on local ideas about race. I initially focused on sickle-cell disease, followed by high blood pressure, so as to include within my research both a genetic and a multi-factorial condition. During this research I came to realize that both biomedical professionals and patients alike used, at times concomitantly and at times alternately, several ideas about race, bodily difference, genetics, and historical processes, combining discourses that could be seen as stemming from different moments of Brazil's history (reference removed by the author). Take for example one of the cardiologists that I followed during my research. On one occasion, while I was observing consultations with patients, he received a visit from a pharmaceutical sales representative. She handed the doctor, amongst several samples of anti-hypertensive medications, a magazine targeted at medical professionals, which he promptly gave me while he walked her out of the consultation room. On the magazine cover was a headline for one of the stories inside on race and hypertension. This doctor had previously made a comment about how Bahia was the land where “everyone was black, everyone has some mixture.” After the pharmaceutical sales representative's visit, and my questioning the cardiologist about the article in the magazine, he started pointing out to me some patients whose blood pressure was harder to get under control “because of their (the patient's) race.” Invariably, these patients had very dark skin. When I asked him if the same logic applied to lighter skinned patients he argued that it could also be the case, “if they carried the gene.” However, he added “the darker (a patient), the more difficult it is to control their blood pressure, in terms of group, obviously, in statistical terms.” What about white patients? “In their case it is not too bad, in those of Spanish, Portuguese ancestry, but you see that here in Bahia there aren't whites…there's mixture already” (Calvo-González, 2011).

This cardiologist discursively combined elements from 19th century ideas about purity, both applied to “whiteness” and “blackness,” with a general view of the local population as admixed. With regards to “whiteness,” at times he used a more encompassing ideas about “whiteness,” which included those individuals of admixed ancestry, who were also passive to having the same bodily characteristics as outwardly looking “blacks” with regards to high blood pressure. At others, he considered to be “real whites” only those who had direct and exclusive European ancestry. With regards to “blackness,” he also oscillated into considering all population admixed and therefore “non-white,” while incorporating at others explicit historical reading of the origins and processes that constituted the Brazilian population with regards to the “purity” of local “blacks.” He once told me that the Portuguese colonizers brought two types of slaves, bantus, and nagôs: “The nagôs were taller, the bantus shorter, thicker. And you saw that nearly all of the blacks whose blood pressure was difficult to control were tall, did you notice that?” I might have not been able to disguise the look of disbelief on my face, which he interpreted as my doubting what he was arguing: “(…) the only thing is that later on there were all those processes of admixing and this makes it harder to control our samples, doesn't it?” This reading of difference between Black bodies echoes Rodrigues' (1932) 19th century writings, in which slaves brought from Western Africa (nagôs) were considered to be physically and culturally superior to slaves brought from Central Africa (bantus).

In spite of these racialized considerations, I never saw that cardiologist, during his clinical practice, employing race as a factor in deciding a diagnosis or treatment protocol. The link he made between disease and race was therefore more of a discursive, rhetorical link between past and present, between contemporary bodies present at the clinic (more specifically, patients with a very dark skin complexion), and bodies from within Brazil's history. In the process, he mobilized ideas about normalcy, not in the sense of establishing a standard for what Black bodies are, but to establish a narrative about what is a normal, as in average, Bahian.

## Racial Difference Implies Different Reference Values? White Blood Cell Count and Normalcy

The ways in which normalcy and race were entangled was made even clearer when I received an invitation to participate in an event titled “Ethnic issues and reference values for clinical laboratory tests.” This meeting was organized in November 2007 by the Immunology and Molecular Biology Center, of the Federal University of Bahia's Institute for Health Studies. As a postdoctoral researcher at the Center for Afro-Asian Studies, of the same institution, I participated alongside another anthropologist, a geneticist, two biologists, two activists from the field of Black Population Health, from within the ranks of the organized Black Social Movement, as well as several students from the Immunology postgraduate programme. The aim of the meeting, as presented by one of its organizers, was to discuss the need to establish new reference values, based on ethnic^7^ groups, for some of the common laboratory tests routinely used by doctors in Bahia, amongst which was the complete blood count. After a short presentation on ancestry markers and the levels of admixture present in the Brazilian population by one of the leading population geneticists of Bahia, a forum for discussion was opened. One of the two Black Population Health activists present at the event, who also worked for the local municipal government, argued for the need to change the reference values for laboratory tests currently used in Bahian medical establishments. According to her, the existing values were established for white patients, overlooking the specificities of the local population. It seemed, at first sight, that the need to adjust these reference values was a consensus amongst all seminar participants. But what did they mean by “local population specificities?” One of the biochemists expanded on this in her presentation, referring to a situation similar to the one described at the beginning of this article. She pointed out how current reference values meant that the laboratory blood analysis results for many local patients were considered abnormal when, in fact, they were absolutely healthy, even when outside of the normal reference range. She explained that these reference values were based on studies undertaken by pharmaceutical companies in Europe and the United States using a sample of white individuals, a “racial” profile that was not adequate for the local population. I asked whether there were local studies on which to base the argument that existing reference values were inadequate for the “local Bahian population.” The biochemist pointed out that there was still no data available, although research was being undertaken to provide this information. Even though it was not directly mentioned by any participant at the event, the reasoning behind the need to review white blood cell reference values for “blacks” was that the international biomedical literature suggested the existence of lower white blood cell counts amongst 25–50% of people of African descent (Haddy et al., 1999), a condition that is not associated with any increased risk for infections or other clinical manifestations termed benign ethnic neutropenia.

Two unspoken assumptions seemed to be at stake: the idea that existing reference values were adequate enough to be applied to local “whites,” but not to local “non-whites,” and the association between “local population specificities” and “non-white.” In this sense, race was a universal link between all bodies identified as white and all those identified as Black, regardless of their ancestry or country of origin. This association would become clearer in 2010, when the results of the research project the biochemist mentioned were published. In a paper written in English and published in a Brazilian journal, the results of a sample of 289 blood donors from Bahia, of which 49.5% self-identified as Black, found no statistical differences in white blood-cell values between different ethnic groups (Felix et al., 2010). The results suggested that “no specific genetic structure is present in the population which is attributable to a high level of heterogeneity.” The authors' conclusion was that establishing different reference ranges for different ethnic groups would not, in fact, be helpful, given that “all individuals present a degree of admixture.” In light of these results, the “local population specificities” mentioned during the 2007 event would in fact apply to all participants in that study, including self-classified “whites.”

When it comes to how race featured in that article, three different categories were used: ethnicity in the article's abstract, “blackness” when referring to how participants self-described their race, and African-Americans when comparing the results with other studies on the same topic. These categories were presented as if they were interchangeable, even though the authors explicitly pointed out that the profile of the local population in terms of white blood cell count was closer to that found by studies undertaken amongst African-Americans than the profile of participants in studies carried out in African countries such as Uganda. This was interpreted by the authors as stemming from the high level of admixture of African-Americans and of Bahia's population.

## Justice, Racial Inequality, and the Politics of Difference

This brings me to another issue that caught my attention in that paper, which I also noticed during the seminar: the marked absence of a discussion on intermediate racial categories. It is worth noting that the hematology unit where the participants for the above mentioned study were recruited, collects data on racial self-classification using the official current census categories (black, brown, white, yellow, and indigenous). Although there is no discussion about how individual's race was collected, presumably the researchers used the information already gathered by the institution and then added up the self-declared Black (*pretos*) and “brown” (*pardos*) individuals to make up the Black group. During the seminar, I initially interpreted this lack of mention of intermediate categories as a result of how the discussion focused on data arising from international biomedical literature that advocates for the implementation of specific racial values for white blood cell counts. This literature tends to focus on “blacks,” African-Americans or Africans, so I presumed the use of the category “non-white” by participants in the seminar was a way of summing up these categories into one that applied to all these groups. The absence of intermediate categories was only timidly questioned by one of the immunology students at the seminar, who questioned the association, made by the other anthropologist between poverty and “blackness” in Bahia. The student questioned whether all those that could not be classified as “whites” would consider themselves to be Black, but no one took up this issue for further discussion. It was when poverty was being discussed that it became clearer to me that the absence of intermediate racial categories was related to a political discourse on racial inequality and the need to reduce such inequalities and effect social justice through affirmative action policies. This was clear from the interventions of all Black Health activists who were participating in the seminar. As I pointed out before, organized Black social movements have consistently highlighted the need to dismantle the racist underpinnings of a society in which those who self-declare as Black and “brown” have a statistically lower life expectancy, are victims of violence in higher numbers (including State violence), earn less, and have less access to health and education services than their white or “yellow” counterparts^8^. The struggle to undo these inequalities often goes hand in hand with the need to unmask the role that the myth of racial democracy had in maintaining racist practices, be they individual or institutional. In that sense, the struggle against racism and for social justice were intertwined with the opposition to the use of intermediate categories, seen as hiding the true experience of racism, as underlined by statistical data, that non-whites suffer, even when their skin is lighter. As such, for many activists, fighting for a more equal society meant dropping the use of intermediate racial categories at certain key moments, at the very least discursively.

The discussion on the need for establishing racial values for white-cell count tackled the possibility of redressing social injustice in a twofold way. On the one hand, addressing the specificities of “blacks” in terms of healthcare was on the agenda of the organized Black Social movement in Bahia. According to activists, these specificities, including the lack of access to services or the neglect at a policy and individual treatment level of some diseases that affected mainly the “black population,” such as sickle-cell anemia, were not taken into account when designing and implementing health policies^9^. On the other hand, race was often used by industries as an argument to dismiss workers' claims of having suffered toxic exposure to chemicals such as benzene, causing leukemia manifested in its early symptoms by a low white-cell count. Sociologist Correia (1998) narrates a situation in which a worker, affected by benzene intoxication and consequently suffering from bone-marrow cancer, had his medical claim of having been a victim of exposure to chemicals during his work dismissed by the company doctor, who told him that his low white blood cell count was due to his being “Bahian, being brown, black.” As a result of situations like this, and to avoid future legal issues related to workers' health, many industries decided not to hire individuals whose pre-admission laboratory tests pointed toward a pre-existing low white blood cell count, even when it was not indicative of any malignant condition. Activists considered that establishing reference values that took into account these normal variations and that allowed for a wider range of reference values would help prevent these situations from arising, by making it more straightforward to differentiate non-malignant low white-cell counts from those that were indicative of an underlying disease.

## Conclusion: Normalcy and the Layering of Discourses on Race

In one of those ethnographic coincidences on which we anthropologists, thrive, a month after the seminar under discussion I witnessed another scene that highlighted these two features of race and normalcy coming together. I was observing one of the first assessment visits, in which hematologists see patients who have been referred to the clinic. A patient came into the room with a complete blood count result with relatively low levels of neutrophils, a particular white cell type. The doctor looked at the patient's results and quickly reassured her. “Don't worry about those neutrophils of yours. I also have low levels; it's normal amongst the Black Population.” The patient, with the exact same skin color as the doctor, an intermediate tone, looked at her with an expression that was a mixture of surprise and disbelief, and gave a half-smile to the woman who had accompanied her into the consultation, which the doctor interpreted as a questioning of her having self-described as Black. “It might not look like it, but I also have a foot in the kitchen,” said the doctor, using a common expression to informally acknowledge African ancestry. Immediately after, the doctor left the room to fetch a document related to the patient's record. The patient turned around and, pointing at a long hair on her own arm, whispered with a mischievous look on her face to her companion: “this is caused by mixture. Crazy things like this happen. Did you hear what the doctor said?” With this comment, the patient was not questioning the association between race and low white-cell count, but subverted the doctor's use of the bipolar system of classification by arguing that the condition was a result of a characteristic commonly seen as defining Brazil's population: admixture.

Taken both at a population and individual bodily level, normalcy serves to contrast local Brazilian history, portrayed as the result of widespread admixture between groups, with other national contexts, such as that of the United States. On the one hand, what is seen as normal for contemporary Brazil is a material body that cannot be classified as pure. On the other, purity features discursively as either existing in a long lost past of the country's own history or in the present context of other countries, such as the United States, identified as the place where the correlation in biomedical studies between race and disease is established^10^. At the same time, these ideas of normalcy can also be locally challenged by certain actors, such as social movement activists, who highlight the specific experience of certain racialized bodies, questioning the overarching national narratives of admixture and arguing for the need to recognize these bodies as also being normal, particularly in the context of political struggle for the reduction of social inequality. These two ways in which normalcy appears connected with local meanings of race give way to seemingly contradictory and opposing discourses: racial categories that are explicitly not identified with admixture are both seen as a thing of the past (the history of the Nation) and a thing of the future (a more equal country), a contradiction that can be better understood through the process through which different historical discourses on racial difference in Brazil are not wholly substituted by one another, but are rather layered and incorporated into a complex weaving of ideas about purity and admixture. In this layering of meanings about race, M'charek (2014) idea about the “folded object” can help us understand how various entities, from diverse moments of history and locations, can be included in a single object. Looking at how objects and practices around them enact race, she shows how race moves and changes shape depending on the times and places in which these objects are deployed. Following M'charek, biomedical technologies, such as complete blood count, can at times “unfold” and bring out racial ideas originally arising in different times and locations. These ideas can include 19th and 20th century ideas about purity and pure racial types, notions about what makes the Brazilian population different, terms from classificatory categories from other countries, such as the United States, discourses on ethnicity vs. race, or the political struggle to reduce inequalities.

The notion of normalcy, within this context, serves as a link between biomedical practices and discourses and ideas about difference embedded within bodies, both at an individual and population level. Although race often seems to appear as a straightforward category to classify biological difference, on-the-ground ethnographic analyses of biomedical practices can show how it appears in connection with, or as a proxy for, ideas such as nation, population or gender, originating from different places and moments in history. Showing how these connections between time, place, objects and discourses operate locally can help us understand the processes through which race sustains its durability as a category through which norms and values related to bodily difference and deviation, characterized as benign or serious pathologies, are conceptualized and naturalized.

## Ethics Statement

The project was approved by the Comitê de Ética em Pesquisa FiocruzCentro de Pesquisas Gonçalo Muniz-CPQGM (93/2006). Written informed consent was obtained from participants in the study as approved by the Ethics Committee. Participation in the seminar was not examined by the Ethics Committee as it was an open event held in public university premises. Ethics approval for this was not required as per applicable institutional and national guidelines. Oral informed consent was obtained from seminar participants.

## Author Contributions

The research and writing up of the article was done by EC-G.

### Conflict of Interest

The author declares that the research was conducted in the absence of any commercial or financial relationships that could be construed as a potential conflict of interest.

## Footnotes

^1^Vigorous exercise is known to temporarily raise white-cell count.

^2^For a classical, very detailed analysis of how this process of measuring and establishing comparisons between “races” was fraught with preconceived ideas about the superiority of “whites” see Gould (1996).

^3^Differences in disease susceptibility were employed in some countries such as the United States to justify migratory restrictions that favored Northern European immigrants against other groups (Markel and Stern, 2002). Another central issue at stake was, particularly with regards to “blacks,” proving how they were physiologically and psychologically suited to, and even inclined toward, being enslaved (Guillory, 1968).

^4^Exemplified in the absence of anti-miscegenation laws.

^5^Most institutions employed self-declaration of candidates as “black” to identify beneficiaries, although some required candidates to submit a photograph to have their self-declaration corroborated by a commission (Maio and Santos, 2005).

^6^Another controversial issue was the way in which the inclusion of the intermediate category within the “black” group could be seen as erasing indigenous ancestry from Brazil's history and racial landscape, or what de Carvalho (2004) termed the “statistical genocide” of this ancestry.

^7^The term é*tnico (*ethnic) is often used in Bahia interchangeably to mean race, which was the case in that event. It is also at times used in a hyphenated combination: é*tnico-racial*.

^8^Indigenous people fare as badly as “blacks” and “browns” in terms of welfare indicators, but although they are sometimes included alongside “blacks” in affirmative action policies, they are often left out of debates on racial inequality in Brazil.

^9^As a result of this activism, successful initiatives such as programmes directed at diagnosis and treatment of sickle-cell disease were established at a local, regional, and national level.

^10^Very similar discourses on race, purity, and admixture are deployed by contemporary geneticists in Brazil. See Wade (2017) and Wade et al. (2014) for detailed analysis of these discourses and how they are also crossed over with long-standing ideas about Nation and population.

## References

[B1] Braun L. (2014). Breathing Race Into the Machine: The Surprising Career of the Spirometer From Plantation to Genetics. Minneapolis, MN: University of Minnesota Press. 10.5749/minnesota/9780816683574.001.0001

[B2] Calvo-González E. (2011). Construindo corpos nas consultas médicas: uma etnografia sobre hipertensão arterial em Salvador, Bahia. Caderno CRH 24, 81–96. 10.1590/S0103-49792011000100006

[B3] Canguilhem G. (1989). The Normal and the Pathological. New York, NY: Zone Book.

[B4] Correia L. (1998). A Epidemia de um Estigma: Leucopenia e Benzenismo entre trabalhadores do Pólo Petroquímico de Camaçari, Bahia (Dissertação Mestrado). Universidade Federal da Bahia, Salvador, Brazil.

[B5] de Carvalho J. M. (2004). Genocídio racial estatístico. O Globo, 27, p. 7.

[B6] Degler C. N. (1971). Neither Black Nor White: Slavery and Race Relations in Brazil and the United States. Madison, WI: University of Wisconsin Press.

[B7] Felix G. E. Abe-Sandes K. Bonfim T. M. Bendicho M. T. Cisneiros P. Guedes R.. (2010). Ancestry informative markers and complete blood count parameters in Brazilian blood donors. Rev. Bras. Hematol. Hemoter. 32, 282–285. 10.1590/S1516-84842010005000074

[B8] Fry P. H. (2005). O significado da anemia falciforme no contexto da 'política racial' do governo brasileiro 1995-2004. Hist. Ciênc. Saúde Manguinhos 12, 347–370. 10.1590/S0104-59702005000200007 16353330

[B9] Gould S. J. (1996). The Mismeasure of Man. New York, NY: WW Norton and Company.

[B10] Guillory J. D. (1968). The pro-slavery arguments of Dr. Samuel A. Cartwright. La Hist. 9, 209–227. 11635310

[B11] Guimarães A. (1999). Raça e os estudos de relações raciais no Brasil. Novos Estudos. CEBRAP 54, 147–156.

[B12] Haddy T. B. Rana S. R. Castro O. (1999). Benign ethnic neutropenia: what is a normal absolute neutrophil count? J. Lab. Clin. Med. 133, 15–22. 10.1053/lc.1999.v133.a94931 10385477

[B13] Harris M. (1956). Town and Country in Brazil, Vol. 37. New York, NY: Columbia University Press.

[B14] Hasenbalg C. (1979). Discriminação e Desigualdades Raciais no Brasil. Rio de Janeiro: Edições Graal.

[B15] Maio M. C. Santos R. V. (2005). Política de cotas raciais, os “olhos da sociedade” e os usos da antropologia: o caso do vestibular da Universidade de Brasília (UnB). Horiz. Antropol. 11, 181–214. 10.1590/S0104-71832005000100011

[B16] Markel H. Stern A. M. (2002). The foreignness of germs: the persistent association of immigrants and disease in American society. Milbank Q. 80, 757–788. 10.1111/1468-0009.00030 12532646 PMC2690128

[B17] M'charek A. (2013). Beyond fact or fiction: on the materiality of race in practice. Cult. Anthropol. 28, 420–442. 10.1111/cuan.12012

[B18] M'charek A. (2014). Race, time and folded objects: the HeLa error. Theory Cult. Soc. 31, 29–56. 10.1177/0263276413501704

[B19] Nogueira O. (1955). Preconceito racial de marca e preconceito racial de origem. Tempo Soc. Rev. Sociol. USP 19, 287–308.

[B20] Pierson D. (1971). Brancos e Pretos na Bahia: Estudo de Contacto Racial, Vol. 241. São Paulo: Companhia editora nacional.

[B21] Pinto L. A. C. (1953). O Negro no Rio de Janeiro. São Paulo: Companhia Editora Nacional.

[B22] Piza E. Rosemberg F. (1999). Cor nos censos brasileiros. Rev. USP 40, 122–137. 10.11606/issn.2316-9036.v0i40p122-137

[B23] Pollock A. (2012). Medicating Race: Heart Disease and Durable Preoccupations With Difference. Durham, NC: Duke University Press. 10.1215/9780822395782

[B24] Rodrigues R. N. (1932). Os africanos no Brasil. Rio de Janeiro: Companhia Editora Nacional.

[B25] Sansone L. (1996). ‘Nem somente preto ou negro': o sistema de classificação racial no Brasil que muda. Afro Ásia 18, 165–187.

[B26] Sansone L. (2004). Negritude sem etnicidade: o local e o global nas relações raciais e na produção cultural negra do Brasil. Salvador: Edufba.

[B27] Santos J. T. (2005). O Poder da Cultura e a Cultura no Poder: A Disputa Simbólica da Herança Cultural Negra no Brasil. Salvador: Edufba. 10.7476/9788523208950

[B28] Stepan N. (1991). The Hour of Eugenics: Race, Gender, and Nation in Latin America. Ithaca, NY: Cornell University Press.

[B29] Wade P. (2017). Degrees of Mixture, Degrees of Freedom: Genomics, Multiculturalism, and Race in Latin America. Durham, NC: Duke University Press. 10.1215/9780822373070

[B30] Wade P. Lopez-Beltran C. Restrepo E. Santos R. V. (eds.). (2014). Mestizo Genomics: Race Mixture, Nation, and Science in Latin America. Durham, NC: Duke University Press. 10.1215/9780822376729

[B31] Wailoo K. (1999). Drawing Blood: Technology and Disease Identity in Twentieth-Century America. Baltimore: Johns Hopkins University Press.

